# Neutrophils drive pulmonary vascular leakage in MHV-1 infection of susceptible A/J mice

**DOI:** 10.3389/fimmu.2022.1089064

**Published:** 2023-01-06

**Authors:** Henry H. Gong, Matthew J. Worley, Kyle A. Carver, Daniel R. Goldstein, Jane C. Deng

**Affiliations:** ^1^ University of Michigan, Ann Arbor, MI, United States; ^2^ Research Service, Veterans Affairs (VA) Ann Arbor Healthcare System, Department of Veterans Affairs Health System, Ann Arbor, MI, United States; ^3^ Division of Pulmonary and Critical Care Medicine, Department of Internal Medicine, University of Michigan, Ann Arbor, MI, United States; ^4^ Division of Cardiology, Department of Internal Medicine, University of Michigan, Ann Arbor, MI, United States; ^5^ Medicine Service, Veterans Affairs (VA) Ann Arbor Healthcare System, Department of Veterans Affairs Health System, Ann Arbor, MI, United States

**Keywords:** neutrophils, coronavirus, viral pneumonia, acute lung injury, murine hepatitis virus, pulmonary vascular leakage

## Abstract

**Background:**

Lung inflammation, neutrophil infiltration, and pulmonary vascular leakage are pathological hallmarks of acute respiratory distress syndrome (ARDS) which can lethally complicate respiratory viral infections. Despite similar comorbidities, however, infections in some patients may be asymptomatic while others develop ARDS as seen with severe acute respiratory syndrome coronavirus 2 (SARS-CoV-2) infections for example.

**Methods:**

In this study, we infected resistant C57BL/6 and susceptible A/J strains of mice with pulmonary administration of murine hepatitis virus strain 1 (MHV-1) to determine mechanisms underlying susceptibility to pulmonary vascular leakage in a respiratory coronavirus infection model.

**Results:**

A/J animals displayed increased lung injury parameters, pulmonary neutrophil influx, and deficient recruitment of other leukocytes early in the infection. Moreover, under basal conditions, A/J neutrophils overexpressed primary granule protein genes for myeloperoxidase and multiple serine proteases. During infection, myeloperoxidase and elastase protein were released in the bronchoalveolar spaces at higher concentrations compared to C57BL/6 mice. In contrast, genes from other granule types were not differentially expressed between these 2 strains. We found that depletion of neutrophils led to mitigation of lung injury in infected A/J mice while having no effect in the C57BL/6 mice, demonstrating that an altered neutrophil phenotype and recruitment profile is a major driver of lung immunopathology in susceptible mice.

**Conclusions:**

These results suggest that host susceptibility to pulmonary coronaviral infections may be governed in part by underlying differences in neutrophil phenotypes, which can vary between mice strains, through mechanisms involving primary granule proteins as mediators of neutrophil-driven lung injury.

## Introduction

Coronaviruses are positive-sense RNA viruses that infect a large range of animals, including humans, in whom they typically cause respiratory infections including the common cold. Viruses of the *Betacoronavirus* genus, however, have demonstrated the potential to cause severe disease and epidemics. This genus includes the epidemic species severe acute respiratory syndrome coronavirus (SARS-CoV)-1 and -2, and Middle East respiratory syndrome coronavirus (MERS-CoV). The ongoing COVID-19 pandemic caused by SARS-CoV-2 has killed millions of people globally and emphasizes the need to better understand the pathophysiology of *Betacoronavirus* infection, which is characterized by immune dysregulation and rapid onset of acute respiratory distress syndrome (ARDS) in epidemic strains (1, 2). Initially identified in 1949 as an encephalomyelitic murine virus, the Murine Hepatitis Virus (MHV) is the most studied coronavirus model for human infection and presents with a variety of strain-specific tropisms (3, 4). The MHV strain 1 (MHV-1) is highly pneumotropic. In susceptible mouse strains such as A/J mice, MHV-1 has been shown to produce the histopathological hallmarks of diffuse alveolar damage, including hyalinization and pulmonary edema, that is characteristic of ARDS during SARS and COVID-19 in humans (5–10).

Although MHV-1 lung infection in A/J mice demonstrates intense inflammatory infiltration, interstitial pneumonitis, and fibrin deposition, C57BL/6 (B6) mice are notably resistant to MHV-1 infection, and the mechanisms underlying the strain-specific disparities in this infection model are not fully understood (5). In human COVID-19 cases, altered neutrophil recruitment, particularly increased neutrophil-lymphocyte ratios, has been shown to be strongly correlated with progression of severe disease with neutrophils dominating the bronchoalveolar spaces of mechanically ventilated patients (11–13). Enhanced neutrophil activation profiles strongly predict critical COVID-19 illness, and severe patients present with higher plasma levels of neutrophil effectors such as neutrophil extracellular traps (NETs) and granule proteins such as elastase, myeloperoxidase (MPO), and cathepsin (14–16). However, neutrophils also play a role in viral killing and clearance of virus-infected cells (17, 18). Thus, the exact role of neutrophils in the pathogenesis of severe respiratory viral infections has been difficult to elucidate.

To address the role of neutrophils in respiratory coronavirus infection, we used the MHV-1 infection model of susceptible A/J and resistant B6 mice. We found that neutrophils are required for extensive pulmonary hyperpermeability that is characteristic of early stages of infection in A/J mice. Compared to the resistant B6, susceptible mice exhibit a relatively elevated ratio of neutrophils to lymphocytes prior to the onset of mortality, with a relative paucity of B cells, macrophages, NK cells, and T cells. In addition, gene and protein analysis of bone marrow neutrophils reveal a marked and constitutive increased expression of primary granule proteins in the A/J mice relative to B6 mice. These proteins are released in higher concentrations in bronchoalveolar lavage (BAL) fluid in infected mice and may mediate heightened pulmonary tissue damage that contributes to respiratory failure and ultimately death.

## Methods

### Mice

C57BL/6 and A/J mice were obtained from Jackson Laboratory and bred in-house at the Ann Arbor Veterans Affairs Medical Center and provided with food and water *ad libitum*. Mice were housed in specific-pathogen free conditions in microisolator cages. Mice were 14-24 weeks of age and were age and sex-matched for each experiment. All experiments were approved by the Veterans Affairs Institutional Animal Care and Use Committee and were performed in accordance with NIH guidelines.

### Virus and infection

Murine Hepatitis Virus-1 was obtained from the American Type Culture Collection (ATCC VR-261) and expanded through passage in L2 cells (ATCC CCL-149) followed by lysis of cells by freeze thaw after 24 hours of infection. Mice were infected with either 2500 PFU or 5000 PFU of MHV-1 diluted in sterile phosphate-buffered saline (PBS) in 30µL volume *via* intratracheal (i.t.) instillation as previously described (19). Mice were monitored daily for weight and disease symptoms and were euthanized if weight loss exceeded 30% of day 2 post-infection (PI) weight or if a combination of weight loss and clinical symptoms met humane euthanasia criteria.

### Depletion of neutrophils

Mice were treated intraperitoneally (i.p.) with 300 µg anti-Ly6G antibody (clone: 1A8 BioXCell BP0075-1) or an isotype control (clone: 2A3 BioXCell BP0089) diluted in in 100µL PBS every other day starting 1 day before infection. Depletion of >90% of neutrophils was validated following euthanasia in lung *via* flow cytometric analysis of CD11b+, Ly6C+, F4/80- cell population, and depletion was further validated by MPO protein measurements in BAL fluid (20, 21).

### Bronchoalveolar lavage

Mice were euthanized and trachea were intubated with 23 gauge polyethylene tubing attached to a syringe. Lungs were inflated with 1mL PBS and 5mM EDTA (Invitrogen 15575-038) and fluid was then withdrawn after gentle palpation of the lung. Cells were pelleted at 500g for 5 minutes and the BAL supernatant was retained for protein analysis.

### Lung digest

Mice were perfused with PBS and 5mM EDTA. Lungs were collected and minced before digestion in RPMI medium containing 5% fetal bovine serum (FBS) with 267.8U/mL collagenase IV (Worthington Biochemical CLSS-4) and 50U/mL DNase I (Sigma-Aldrich D4263) at 37°C. Lung digests were repeatedly pressed through a 18-gauge needle before being passed through a 70um cell strainer. Blood cells were lysed with 2mL ammonium-chloride-potassium (ACK) buffer for 2min on ice. Leukocytes were then enriched through centrifugation in 20% HBSS-buffered Percoll (Cytiva 17544501). Following resuspension cells were counted and used for flow cytometry.

### Flow cytometry

Isolated cells were stained with Zombie Aqua fixable viability dye (BioLegend 423101) and washed in PBS. Fc receptors were blocked with anti-CD16/32 in 2% FBS-PBS. Cells were then stained with antibodies for desired surface markers. Following staining, cells were washed and fixed in 4% paraformaldehyde buffer (Biolegend 420801). Cells were acquired on an LSRII cytometer (BD) and analyzed using FlowJo 10.8.1 (BD). Leukocyte cell counts were obtained by acquiring total cell numbers post lung digest on a Countess II FL (Thermo Fisher Scientific) and calculating against viability and CD45+ rates acquired through flow cytometric analysis.

### Antibodies

*AF488*: CD45 (clone: 30-F11, BioLegend); *PerCp/Cy5.5:* CD19 (clone: 6D5, BioLegend); *APC*: F4/80 (clone: BM8, BioLegend); *APC-Cy7*: CD4 (clone: GK1.5, BioLegend); *PE-Cy7*: CD8a (clone: 53-6.7, BioLegend); *PE*: CD49b (clone: DX5, BioLegend); *BV650*: CD3 (clone: 17A2, BioLegend); *BV421*: Ly6G (clone: 1A8, BioLegend).

### Neutrophil isolation

To isolate bone marrow neutrophils, mice were euthanized and rear femur and tibia were cleared of skin and muscle tissue in 70% ethanol followed by PBS. Bone marrow was then flushed with 5% FBS RPMI from the femur and tibia with a 27.5g needle. Bone marrow was then flushed through a 20g needle and syringe until broken up. Cell suspension was then passed through a 70um cell strainer. Red blood cells were lysed with 2mL ACK buffer for 2min on ice. To isolate blood neutrophils, blood was collected after euthanasia *via* cardiac puncture with a 23g needle. Red blood cells were lysed with 1mL ACK buffer per 100μL blood collected for 3min on ice. Neutrophils were then isolated using an EasySep PE positive selection kit (STEMCELL Technologies 17666) according to manufacturer instructions using PE-conjugated anti-Ly6G antibody (clone: 1A8, BioLegend).

### PCR

RNA was isolated from bone marrow using an RNeasy Mini kit (Qiagen 74104) according to manufacturer instructions. A High-Capacity cDNA reverse transcription kit (Applied Biosystems 4368814) was used to create cDNA from equivalent quantities of RNA for each sample. RT-PCR was then conducted using a Taqman Gene Expression Assay kit (Applied Biosystems) using GADPH as a housekeeping gene. The relative gene expression of A/J bone marrow neutrophils was then compared against the average gene expression from B6 bone marrow neutrophils.

### ELISA

Elastase, MPO, S100A8, IgM levels in BAL or cell culture samples were measured with ELISA kits (Elastase: R&D systems DY4517; MPO: R&D systems DY3667; S100A8: R&D systems DY3059; IgM: Immunology Consultant Labs E90-M) following manufacturer’s instructions. Cell samples were lysed by suspension in deionized water at a concentration followed by freeze-thaw. Interleukin-1 alpha (IL-1α), interleukin-1 beta (IL-1ß), interleukin-6 (IL-6), interleukin-10 (IL-10), interleukin-12 p70 subunit (IL-12 p70), interleukin-23 (IL-23), interleukin-27 (IL-27), interferon beta (IFNß), interleukin-17A (IL-17A), monocyte chemoattractant protein 1 (MCP-1), tumor necrosis factor alpha (TNF-α), interferon-gamma (IFNγ), and granulocyte macrophage colony stimulating factor (GM-CSF) were measured from lung sonicates using LEGENDplex Mouse Inflammation Panel (BioLegend 740150) according to manufacturer instructions.

### Evans Blue lung permeability assay

At 5 days PI, mice were anesthetized with isoflurane and 500µg of Evans Blue diluted in PBS (5mg/ml) was delivered retro-orbitally. After 30 minutes, mice were euthanized and were perfused through the right ventricle with 10mL of PBS and 5mM EDTA. Lungs were washed in PBS and were then homogenized in 1mL of PBS. A 400µL aliquot was mixed with 800µL of formamide for 18 hours at 60°C. Following dye extraction, samples were centrifuged at 7500g for 10 minutes. Supernatant was transferred into 96-well plate in duplicate with a volume of 200µL. Absorbance was then read at 620nm and 740nm with a Synergy H1 microplate reader (BioTek Instruments). Heme pigment was corrected for according to the following equation: A620_corrected_ = A620 – (1.426*A740+0.030) (22). Total Evans Blue present in the lung parenchyma was calculated using a standard curve.

### Platelet quantification

Mice were euthanized and blood was collected *via* cardiac puncture with a 23g needle and dispensed into a dipotassium EDTA tube (BD 365974). Platelets were then quantified by the University of Michigan *In-Vivo* Animal Core using an Element HT5 Hematology Analyzer (Heska).

### Viral load assessment

Viral load in tissue and in virus-neutrophil coculture supernatants was assessed using plaque assays in L2 cell monolayers. Briefly, L2 cells were grown to 90% confluence and viral samples were added at dilutions ranging from 10^2^-10^5^ in viral growth media (DMEM with 1x penicillin/streptomycin, 3% bovine serum albumin fraction V, and 25mM HEPES). After 24 hours of co-culture, plaques were then counted by eye and plaque-forming units present in original sample were calculated.

### Statistics

GraphPad Prism 9.3.0 (GraphPad Software) was used to calculate significance using Mann-Whitney U tests with false discovery rate approach to correct for multiple comparisons where applicable or with log-rank test for survival. Significant differences are denoted * p<0.05, ** p<0.01, *** p<0.001, **** p<0.0001.

## Results

### A/J mice have a marked susceptibility to MHV-1 respiratory infection

**Figure 1:**
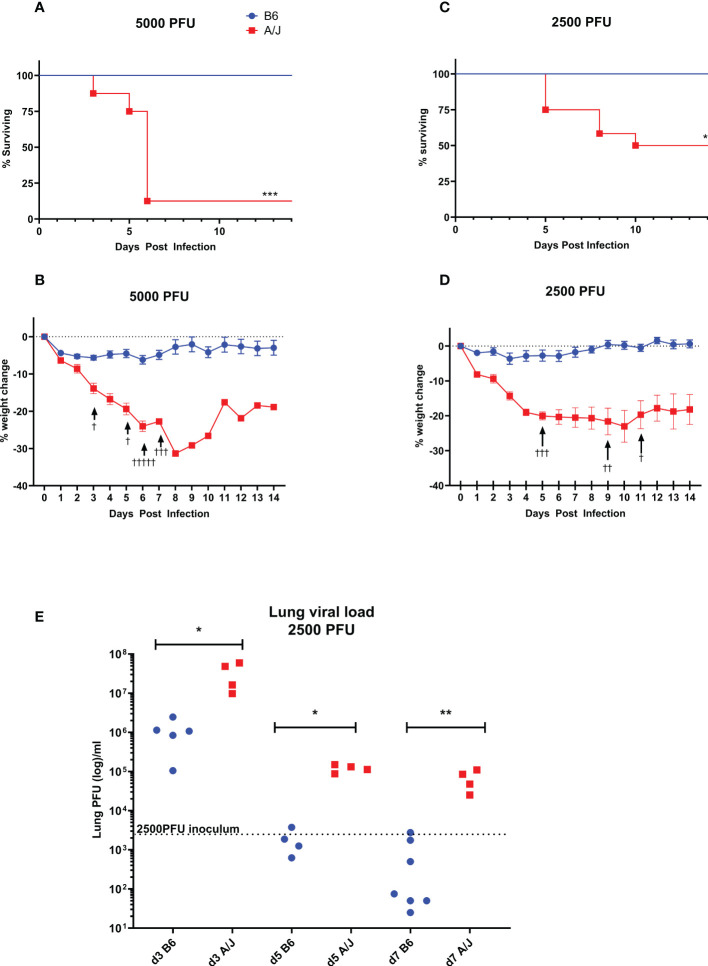
A/J mice are much more susceptible to respiratory MHV-1 infection than B6 mice. A/J and B6 mice were infected intratracheally with either 5000 or 2500 PFU of MHV-1. **(A, B)** The 5000 PFU dose was nearly entirely lethal for A/J mice, with extensive weight loss and mortality beginning at day 3, and most requiring euthanasia by day 7 (93% mortality, n=13). In contrast, all B6 mice survived the dose with minimal weight loss. Data pooled from 2 replicate experiments **(C, D)** The 2500 PFU dose was found to be 50% lethal in A/J mice, with deaths occurring between days 5 and 10 post-infection (n=12). As with the higher dose, weight loss increased dramatically starting on day 3 for A/J mice, while the B6 mice all survived the infection with minimal or no weight loss. Data shown pooled from 2 replicate experiments. **(E)** Lung homogenates were assayed for viral load using a plaque assay in L2 cells on days 3, 5, and 7 post-infection with a 2500 PFU dose. At all timepoints, viral loads in A/J lungs were significantly higher than in B6 lungs, which had effectively cleared virus below inoculum levels by day 5. In contrast, A/J viral loads remained elevated through day 7. † Symbol in A and B denote mouse euthanasia. Statistical analysis for survival by Log-rank test **(A, B)** and for viral load by Mann-Whitney U test **(E)**. Significant differences are denoted *p < 0.05, **p < 0.01, ***p < 0.001.

We sought to model a severe respiratory coronavirus infection with MHV-1 in our A/J mice, which is reported to cause lung pathology reminiscent of SARS in humans in previous studies (5). We found that 5,000 PFU MVH-1 delivered i.t. was 93% lethal to A/J mice (n=13) by day 6 post infection (PI), with the earliest death occurring on day 3 PI (**Figure 1A**). Immediately prior to euthanasia, A/J mice had lost on average 25% of initial bodyweight (**Figure 1B**). In contrast, B6 mice were resistant to the effects of 5,000 PFU MHV-1 with all mice surviving and showing minimal clinical symptoms, with average weight loss on day 7 PI being 5% (**Figures 1A, B**). We found 2500 PFU of MHV-1 was a LD_50_ dose for A/J animals, with deaths occurring between days 5-10 (**Figure 1C**). As with the 5000 PFU dose, the B6 mice all survived the infection and quickly regained all post-operative weight loss (**Figures 1C, D**). To achieve an LD_50_ in B6 mice, a 200-fold higher dose of 500,000 PFU was required, emphasizing the disparity in mortality between strains (**Supplementary Figure 1**).

Based on these results, we assayed the lung viral load with the 2500 PFU inoculum at three timepoints - day 3 as an early infection timepoint, day 5 as the start of the lethal period, and day 7 as the late phase of infection (**Figure 1E**). At all timepoints, the viral burden in A/J mouse lungs was significantly higher than in the B6 mouse lungs. At day 3 PI, the virus had replicated to greater than inoculum levels in both A/J and B6 mice. By day 5 PI, the B6 mice cleared the infection below inoculum levels. In A/J mice, although the viral burden decreased over time, viral levels remained elevated beyond the inoculum dose through day 7, indicating an impaired innate immune response that fails to clear virus from the lungs particularly at early timepoints.

### MHV-1 infection in A/J mice leads to increased vascular leakage

**Figure 2:**
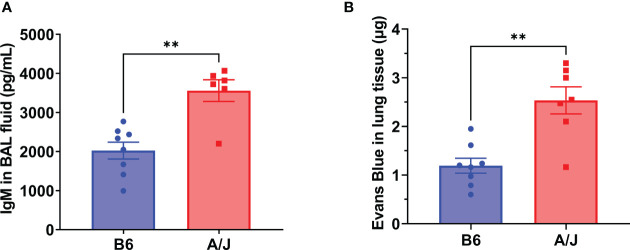
A/J mice exhibit greater lung injury than B6 mice during MHV-1 infection. Vascular permeability was assessed as a marker of lung damage in mice 5 days post-infection with 2500 PFU MHV-1. **(A)** ELISA for IgM from bronchoalveolar lavage fluid showed significantly more leakage into airway spaces in A/J mice compared to B6 mice. Data shown pooled from 2 replicate experiments (n= 8 B6, 6 A/J). **(B)** Following Evans Blue dye delivery, A/J mice displayed significantly higher dye leakage into lung tissue than B6 mice. Data pooled from 2 replicate experiments (n= 8 B6, 7 A/J). Statistical analysis by Mann-Whitney U test for bronchoalveolar IgM **(A)** and tissue Evans Blue levels **(B)**. Significant differences are denoted **p < 0.01.

Severe lung injury is the major cause of mortality from severe respiratory viral infections. Pulmonary epithelial barrier hyperpermeability is a hallmark of acute lung injury, which results from severe infection and inflammation in the lungs (23). To examine the effects of MHV-1 infection on lung damage, we collected bronchoalveolar lavage (BAL) fluid at day 5 PI and assess for elevated immunoglobulin M (IgM) as a measure of alveolar epithelial permeability into the airspaces. We found a significant increase in IgM levels in A/J mice relative to B6 mice, indicating a greater overall degree of tissue damage in the lungs (**Figure 2A**). As a confirmatory assay to demonstrate increased lung epithelial permeability in A/J mice, we delivered Evans Blue intravenously at the day 5 PI timepoint and found a greater penetrance of Evans Blue into the infected A/J mouse lung parenchyma compared to the B6 lungs (**Figure 2B**). Thus, susceptible animals exhibit increased lung injury.

### Innate inflammatory cytokines are increased in A/J lungs compared to B6 lungs during MHV-1 infection

**Figure 3:**
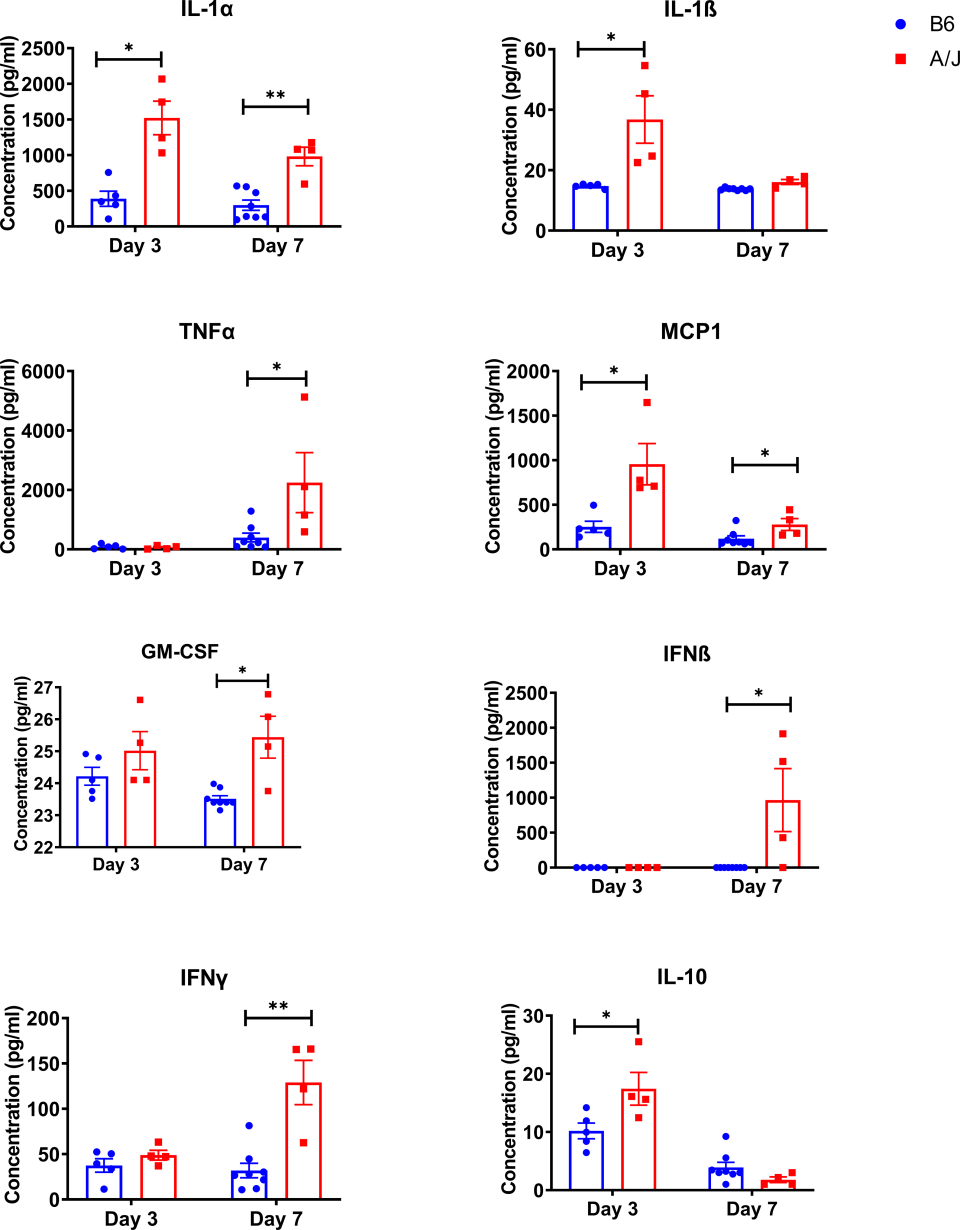
Innate inflammatory cytokines are elevated in MHV-1 infected A/J mouse lungs. Inflammatory cytokines were assayed in lung homogenates from mice 3- and 7-days post-infection with a 13-panel inflammatory cytokine LEGENDPlex immunoassay. Cytokines showing a significant difference between strains at either timepoint are shown (IL-1α d3 and d7, IL-1ß d3, TNFα d7, MCP1 d3 and d7, GM-CSF d7, IFNß d7, IFNγ d7, IL-10 d3). Statistical analysis by Mann-Whitney U test for each cytokine at each timepoint. Significant differences are denoted *p < 0.05, **p < 0.01.

We next assessed whether the increased lung injury in the susceptible A/J strain resulted from an over-exuberant inflammatory immune response (24). Mortality has been shown to correlate better with inflammatory cytokine levels than with viral load in prior studies of coronaviral lung infection in murine models (7, 25). Thus, we sought to determine how inflammatory responses mounted in A/J mice compared to those of B6 animals, particularly in response to changing viral levels over the course of infection. We first measured inflammatory cytokines in lung at days 3 and 7 PI (**Figure 3**). At day 3 PI, there was a significant increase in both IL-1α, IL-1ß, MCP1, and IL-10 in the A/J lungs compared to the B6 lungs. At day 7, IL-1ß, IL-10, and MCP-1 markedly decreased in the A/J group, with IL-1β and IL-10 no longer significantly different compared to B6 animals. In contrast, IL-1α, which is known to be an alarmin released by damaged and necrotic cells (26), remained elevated beyond three-fold in A/J mice compared to B6 mice at both timepoints. IL-1α levels were unchanged between timepoints in B6 mouse lungs, even though MHV-1 had replicated to high viral titers at day 3 PI. Notably, despite the day 3 viral levels in the lungs of B6 animals being 10-fold higher than the day 7 lung burdens of A/J animals, IL-1α levels remained relatively attenuated compared to A/J lung homogenates, indicating a reduced level of tissue damage and alarmin release irrespective of viral replication in B6 lungs.

In addition, we observed significantly elevated levels of TNFα, IFNβ, IFNγ, and GM-CSF in A/J lung homogenates at day 7, despite decreasing viral numbers. Collectively, this suggests that A/J animals have dysregulated inflammatory responses that do not correlate with the kinetics of viral clearance. Given the persistent elevation of multiple inflammatory cytokines in A/J mice, we reasoned there was likely a strong immunologic driver of pulmonary pathology.

### Neutrophil recruitment is increased early in infection in A/J mouse lungs

**Figure 4:**
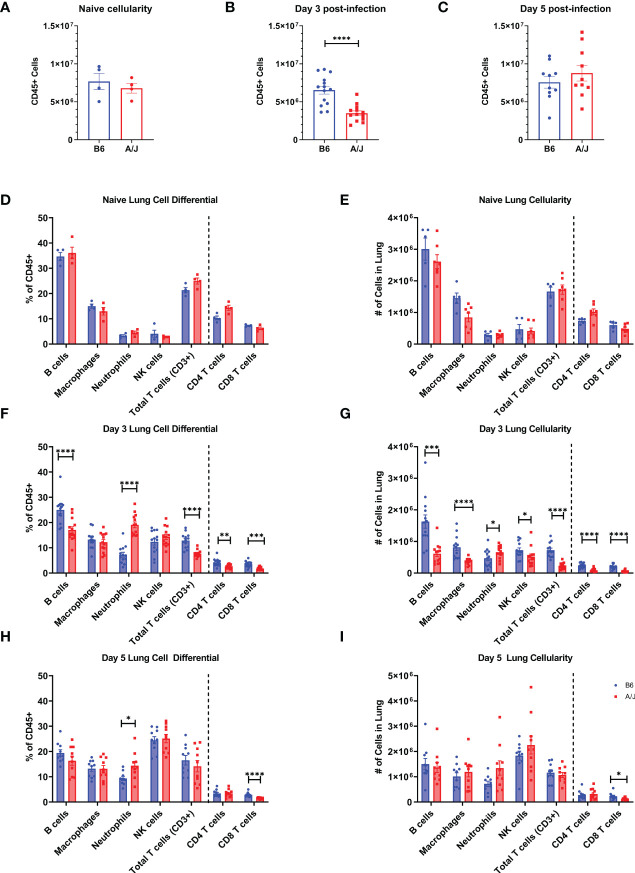
A/J mice have a highly neutrophilic but reduced overall lung leukocyte response early in MHV-1 infection compared to B6 mice. **(A-C)** In naïve lungs, total CD45+ lung cell counts are not different between A/J and B6 mice. At day 3 post-infection, however, the A/J lungs have fewer CD45+ cells compared to B6 mice. By day 5, the cell counts are equivalent between strains. **(D, E)** There are no differences in leukocyte cell type proportions or cell counts in naïve animals. **(F, G)** At day 3, however, A/J mouse lungs have lower proportions of B (CD45+ CD19+) and T (CD45+ CD3+) cells, and a higher proportion of neutrophils (CD45+ Ly6G+). The absolute cell counts of almost all cell populations analyzed, including B cells, T cells, macrophages (CD45+ F4/80+), and NK cells (CD45+ CD49b+) is lower in A/J mice. The total count of neutrophils, however, is higher in A/J mice. **(H, I)** At day 5 PI, the proportions of neutrophils remain elevated in A/J lungs, although to a lesser degree than at day 3 PI and neutrophil cell counts do not differ between strains. The proportion and cell count of CD8 T cells, however, remains lower in A/J lungs compared to the B6 lungs. (Naïve n=4 per strain; D3 PI n=13 per strain; D5 PI n=10 per strain). Statistical analysis by Mann-Whitney U test for bulk CD45+ cellularity **(A-C)** and Mann-Whitney U test with false discovery rate correction (q=0.05) for leukocyte subset cellularity and proportion **(D-I)**. Significant differences are denoted *p < 0.05, **p < 0.01, ***p < 0.001, ****p < 0.0001.

To identify the mediators of lung damage in this model, we next characterized the cellular profiles in the lungs of A/J and B6 mice. AJ and B6 mice received 2500 PFU MHV-1 delivered IT, and leukocyte differentials were performed on the isolated lungs at day 3 and 5 PI. We found no differences in lung leukocyte numbers between naïve mice strains (**Figure 4A**). As neutrophil-platelet interactions are known to promote neutrophil activation and recruitment during infection, we also quantified blood platelet numbers from naïve mice and found no difference in platelet counts (**Supplementary Figure 2**). At day 3, however, we found a significant difference in leukocyte numbers, with less than half as many leukocytes present in the A/J mouse lungs despite the presence of higher viral loads and inflammatory cytokines (**Figure 4B**). At day 5, however, the same number of lung leukocytes were recovered from both strains (**Figure 4C**). Cell differential analysis showed that cell subsets did not vary between strains in the naïve state (**Figures 4D, E**). At day 3, however, B6 lungs contained roughly double the proportion of B cells (26.2%) and T cells (11%) compared to A/J lungs (16.6% B cells and 5.5% T cells). Neutrophils, however, comprised a significantly higher share of cells in A/J lungs (18.7%) which was nearly triple that seen in the B6 lungs (6.6%) (**Figure 4F**). There were significantly fewer absolute leukocyte counts of B cells, T cells, natural killer cells, and macrophages in A/J lungs, while total lung neutrophil counts were higher in A/J mice (**Figure 4G**). Despite the differences at day 3, by day 5 the proportions and counts of most leukocytes had equilibrated between the two strains. Neutrophils remained elevated in A/J lungs, albeit to a much lesser degree compared to day 3 post-infection, and the counts of neutrophils were not significantly different. The proportion and count of CD8+ T cells remained reduced in A/J mice. Curiously, most CD3+ T cells were CD4 or CD8 negative, possibly reflecting an expansion of mucosal γδ T cells (**Figures 4H, I**). Thus, A/J animals appear to display decreased numbers of lymphocyte populations in the lungs during early infection, with a marked elevation in neutrophil numbers compared to their infected B6 counterparts.

### A/J neutrophils express higher levels of primary granule proteins than B6 neutrophils

**Figure 5:**
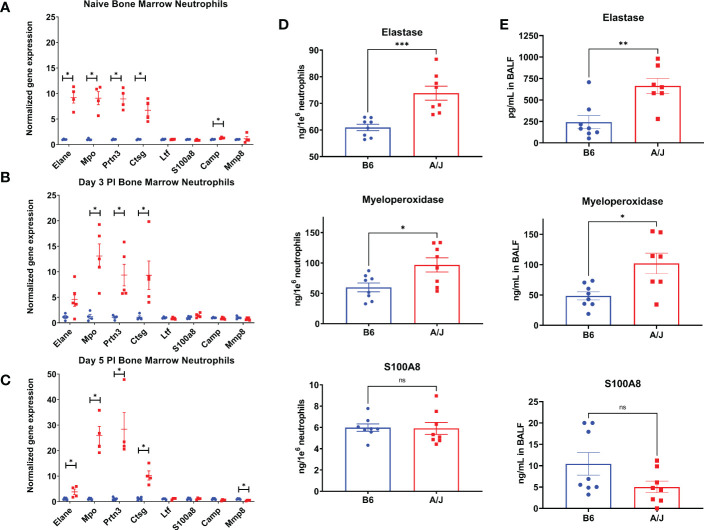
A/J neutrophils express higher levels of primary granule proteins than B6 neutrophils. **(A-C)** The expression of selected genes was measured in bone marrow neutrophils using RT-QPCR. **(A)** In naïve neutrophils, the 4 primary granule protein genes selected – elastase (Elane), myeloperoxidase (Mpo), cathepsin G (Ctsg), proteinase 3 (Prtn3), and cathelicidin antimicrobial peptide (Camp) – were all expressed to a higher degree in A/J neutrophils, while other genes had similar relative expression normalized to GAPDH. **(B)** At day 3, the expression of Mpo, Ctsg, and Prtn3 were all increased in A/J mice. **(C)** At day 5, Elane, Mpo, Ctsg, and Prtn3 gene copies were increased in A/J mice, while matrix metalloproteinase 8 (Mmp8) copies were reduced. **(D)** Elastase, myeloperoxidase, and S100A8 were then measured in supernatants and lysates of bone marrow neutrophils from naïve mice cultured for 6 hours in RPMI. The primary granule proteins elastase and myeloperoxidase were higher in A/J neutrophils, while S100A8, the most highly expressed cytoplasmic neutrophil protein, was equivalent between strains. **(E)** These proteins were measured in bronchoalveolar lavage fluid from mice 5 days post-infection with MHV-1. In alveolar spaces from infected mice, again only elastase and myeloperoxidase were expressed to a greater degree in A/J mice, while S100A8 remained equivalent. Statistical analysis by Mann-Whitney U test with false discovery rate correction (q=0.05) for bone marrow neutrophil gene expression **(A-C)** and Mann-Whitney U test for protein measurements (D,E). Significant differences are denoted *p < 0.05, **p < 0.01, ***p < 0.001 “ns” p>0.05 (not significant).

An excessive neutrophil response has been implicated in lung damage in a variety of viral respiratory infections (27, 28). This tissue injury is thought to occur largely as a result of granule protein release (29–31). We postulated that in addition to the relative increase in numbers of neutrophils in the lungs of infected A/J animals, that there might also be a qualitative difference in granule contents of the neutrophils to explain the increased markers of lung injury. To investigate functional differences between A/J and B6 neutrophils, we isolated neutrophils from the bone marrow using Ly6G positive immunomagnetic selection and assayed for differences in gene and protein expression. RNA was isolated and assayed for gene expression with an RT-PCR panel in bone marrow neutrophils from uninfected mice and 3- and 5-days PI. Interestingly in uninfected mice, A/J bone marrow neutrophils exhibited increased gene expression of all primary granule proteins assayed relative to B6 bone marrow neutrophils with about a 9-fold increase detected in elastase (*Elane*), *Mpo*, and proteinase 3 (*Prtn3*), as well as a nearly 7-fold increase in cathepsin G (*Ctsg*) copies. RNA for cathelicidin antimicrobial peptide (*Camp*), a secondary granule protein, was also increased by about 30% in the A/J neutrophils (**Figure 5A**). At day 3, the genes *Mpo*, *Prtn3*, and *Ctsg* all showed even greater fold-changes in A/J neutrophils over B6 neutrophils compared to the naïve state (**Figure 5B**). At day 5, all primary granule protein genes were again significantly higher in A/J neutrophils, with *Mpo* and *Prtn3* both showing a 25-fold or greater change compared to B6 neutrophils. Matrix metalloproteinase 8 (*Mmp8*) showed about a 2-fold reduction in A/J neutrophils compared to their B6 counterparts (**Figure 5C**). The gene expression of the secondary granule proteins lactoferrin (*Ltf*) and *S100A8* were not different at any timepoint, while differences in *Camp* in naïve mice and *Mmp8* at day 5 post-infection were marginal relative to those detected for primary granule protein genes and likely of limited biological relevance.

To confirm the constitutive enhanced expression of primary granule proteins, we then isolated bone marrow neutrophils from naïve B6 and A/J mice, cultured them for 6 hours in RPMI, and measured the primary granule proteins neutrophil elastase and MPO, and the secondary granule protein S100A8 combined from the supernatant and lysate. In agreement with the gene expression data, neutrophils from uninfected A/J mice produced significantly more elastase and MPO than B6 neutrophils, with a 17.6% increase for elastase and 64.3% increase for MPO, while S100A8 protein quantities remained equivalent between strains (**Figure 5D**). To further confirm these protein differences reflected neutrophil phenotype *in vivo*, MPO was measured from bone marrow neutrophils and blood neutrophils lysed directly after isolation from uninfected mice. Freshly isolated A/J bone marrow neutrophils expressed 27.1% more MPO compared to B6 bone marrow neutrophils (**Supplementary Figure 3A**), while blood neutrophils from A/J mice also expressed 26.9% more MPO (**Supplementary Figure 3B**).

Primary granule proteins are known to be among the most tissue damaging constituents of the neutrophil armament, including a variety of serine proteases and reactive oxygen species generators (32, 33). In addition to the higher numbers of neutrophils present in the lungs of A/J mice, these cells may further compound damage because of the greater presence of these tissue-damaging effector molecules. To investigate whether A/J neutrophils displayed enhanced release of these mediators in the airways during infection, we assayed the presence of neutrophil elastase, MPO, and S100A8 in BAL fluid from mice at day 5 PI when lung neutrophil numbers had equilibrated between the strains. Both neutrophil elastase and MPO levels were significantly higher in BAL fluid from A/J mice, while S100A8 was not significantly different (**Figure 5E**). A/J neutrophils appear to produce and release higher amounts specifically of primary granule proteins in the MHV-1 infected lungs. Combined with the early neutrophilic lung infiltration seen in these mice at day 3 PI, this enhanced primary granule release may heavily contribute to the increased lung tissue permeability seen in these mice.

### Neutrophil depletion ameliorates pulmonary hyperpermeability in A/J mice

**Figure 6:**
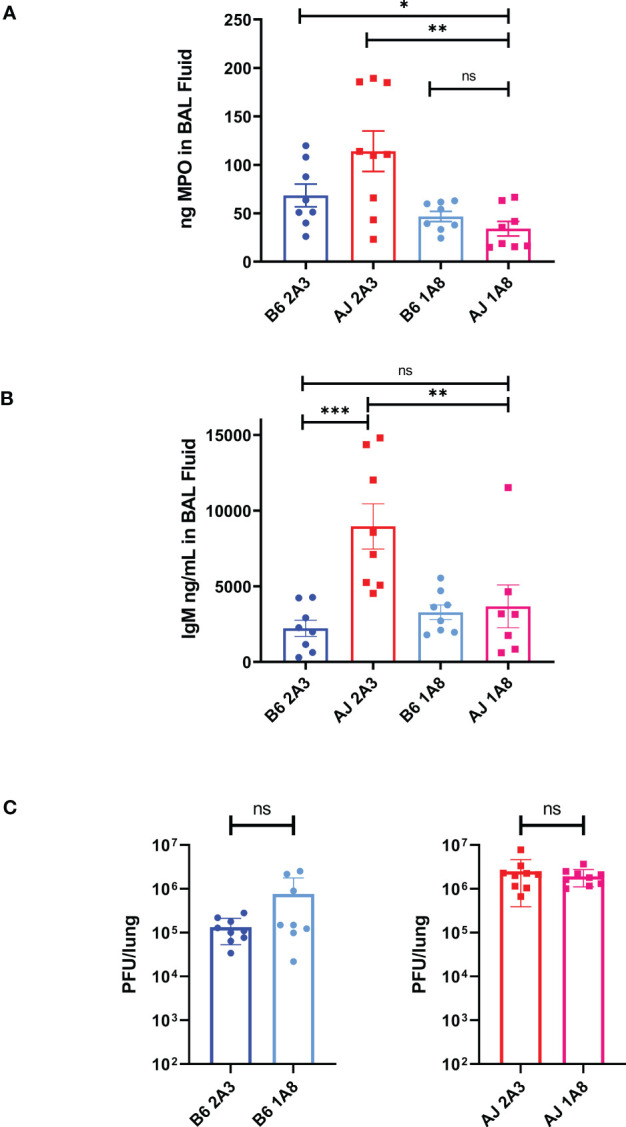
Neutrophils are responsible for the early induction of pulmonary vascular leakage in MHV-1 infected A/J mice. **(A)** MPO was significantly reduced in BAL fluid from 1A8-treated A/J mice compared to 2A3-treated A/J and 2A3-treated B6 mice, showing a reduction in neutrophil activity. **(B)** IgM was measured from BAL fluid samples and 1A8-treated A/J mice had lower levels of IgM compared to isotype antibody-treated A/J mice. IgM levels from 1A8 mice were equal to those found in the B6 mice groups. **(C)** Following neutrophil depletion, viral load was measured via plaque assay from lung samples. Neutrophil depletion had no effect on viral burden in the lungs of either B6 or A/J mice. Data shown are pooled data from 2 replicate experiments. Statistical analysis by Mann-Whitney U tests. Significant differences are denoted *p < 0.05, **p < 0.01, ***p < 0.001; “ns” p>0.05 (not significant).

The relatively elevated proportion of neutrophils and increased primary granule protein expression in A/J mice suggests that neutrophils may be one of the main drivers of lung pathology in A/J mice. To test this, we depleted neutrophils from both strains with anti-Ly6G antibody (1A8 clone) treatments delivered i.p. every other day beginning 1 day prior to infection, with collection of BAL fluid samples for further analysis on day 4 PI, which is just prior to the onset of mortality with a 2500 PFU dose (**Supplementary Figure 4A**). We found a near total depletion (>90%) of Ly6G+/CD11b+ neutrophils in both the lung of both mice when treated with 1A8 antibody compared to mice treated with isotype control 2A3 antibody (**Supplementary Figure 4B**). To ensure a significant depletion of neutrophils and circumvent possible issues with Ly6G membrane downregulation or epitope masking, we measured MPO levels in BAL fluid as an intermediary for neutrophil activity. BAL fluid MPO levels in 1A8-treated A/J mice were significantly reduced compared to both 2A3-treated A/J and B6 mice and equivalent to the concentrations found in 1A8-treated B6 mice (**Figure 6A**). With neutrophil depletion, we found that IgM levels in A/J mice treated with 1A8 antibody decreased to a level equivalent to that of the resistant B6 mice (**Figure 6B**), indicating improved epithelial barrier function. Surprisingly, neutrophil depletion had no effect on lung viral burden, demonstrating that these cells and not increased viral loads are responsible for much of the pulmonary hyperpermeability seen in A/J mice (**Figure 6C**). These results demonstrate that neutrophils are a critical driver of lung vascular damage in MHV-1 infection of susceptible A/J mice, and appear to play little role in viral clearance.

## Discussion

Pulmonary MHV-1 infection of susceptible mouse strains has been proposed as an animal model to study host defense to severe human coronavirus (e.g., SARS-CoV) respiratory infections. In line with previous reports, we found that A/J mice are susceptible to doses that are much lower than those required to establish similar morbidity in the resistant B6 strain. Using a lethal dose (LD_50_) of 2500 PFU MHV-1 delivered intratracheally, we find that similar to patients with severe coronavirus pneumonia, the immune response in infected A/J mice is characterized by relatively elevated neutrophilic proportions in the lungs, along with a heightened innate inflammatory cytokine response. The susceptible A/J strain also exhibit findings consistent with increased pulmonary permeability, reflecting lung injury. We demonstrate that neutrophils are responsible for inducing lung hyperpermeability during MHV-1 infection of the susceptible A/J mouse strain as the depletion of these cells prevents pulmonary vascular leakage induced during the early period of infection. Additionally, the A/J and B6 strains display significant heterogeneity in the neutrophil expression of certain primary granule proteins, starting in the bone marrow compartment and carrying over to the airway spaces. The A/J mouse may provide a model to study the effects of overexpression of these proteins.

Pulmonary epithelial barrier leakage is a hallmark of severe lung injury during many respiratory infections and can result from a combination of pathogen-mediated actions against epithelial and endothelial cells, as well as the immune response against these pathogens (34). Neutrophil influx into the lung parenchyma is often associated with infectious vascular leakage. A wide array of neutrophil granule proteins has been implicated in lung injury, particularly the proteins contained in primary granules including elastase, cathepsin G, proteinase 3, and myeloperoxidase (34, 35). We have found that A/J neutrophils have greater basal levels of gene expression and production of primary granule proteins, and during infection, these mediators are released in the A/J lungs in higher quantities compared to B6 lungs. By depleting neutrophils prior to infection, vascular leakage was reduced in the A/J mouse strain to a level on par with that observed in B6 mice at the day 4 timepoint prior to the onset of mortality. In contrast, neutrophil depletion in B6 mice had no effect on lung permeability, as measured by IgM in the BAL fluid.

We have identified neutrophils as a driver of pulmonary hyperpermeability in MHV-1 infection in A/J mouse strains. As has been reported in human patient populations with severe COVID-19 disease, we observe an increased neutrophil-lymphocyte ratio in the lungs of susceptible mice. Elevated neutrophil-lymphocyte ratios in human patient blood and BAL samples have been reported as prognostic markers for severe SARS and COVID-19 cases, and further study using this model may help uncover the underlying mechanisms and consequences of lymphopenia during severe coronavirus infections (36, 37). Moreover, our results suggest that genotypic differences may induce functional heterogeneity in neutrophils that could predispose individuals to more severe outcomes during coronavirus infection. Even in uninfected A/J mice, bone-marrow neutrophils express increased levels of primary/azurophilic granule genes (e.g., elastase, proteinase 3, cathepsin G, MPO), but not secondary/specific granule genes (e.g., cathelicidin, lactoferrin, MMP8). In addition, over the course of infection, we observed dynamic increases in the difference between A/J and B6 neutrophil gene expression of MPO, proteinase 3, and cathepsin G. The extent to which heterogeneity in neutrophil granule content and composition mediates susceptibility to more severe outcomes following respiratory viral infections in humans is presently unexamined. The underlying genetic determinants of neutrophil granule composition should be studied further for use as a potential determinant of outcome and prognosis. Both MPO and elastase protein, which have also been reported as serum markers of severe COVID-19 infection in humans (14), were upregulated in the airway spaces of A/J mice even after neutrophil numbers had equilibrated at day 5. This finding may reflect increased primary granule protein expression and release as our bone marrow neutrophil gene and protein data suggests, but it may also reflect other strain-specific differences in neutrophil-extrinsic factors. Neutrophil-platelet interactions, neutrophil-endothelial interactions, or neutrophil crosstalk with other leukocytes may differ at steady state between strains and can all modulate neutrophil functions including extravasation, degranulation, and the release of neutrophil extracellular traps (NETs) (38–40). In addition, several primary granule proteins, including myeloperoxidase, elastase, and cathepsin, are well-defined markers of NETs, which have been associated with endothelial barrier disruption and ARDS (41). Accordingly, NETosis has been identified as an important mediator of immunopathology both in the lung and systemically in many respiratory infections including COVID-19, where NETs are known to contribute heavily to lung injury and thrombosis in association with platelet aggregation (42–44). It is possible that the heightened release of primary granule proteins in the airway spaces of A/J mice as well as the increased damage to these areas may result from increased NET release more so than degranulation, and further studies are needed to elucidate this.

Notably, gene expression of non-primary granule proteins, including *Ctsg*, *S100a8*, and *Mmp8*, are reportedly also increased in human leukocytes during severe COVID-19 cases relative to mild cases, but these increases were not seen in murine bone marrow neutrophils in our mild and severe MHV-1 infection model using B6 and A/J mice (45–47). It is important to note that many studies of neutrophil gene expression in severe COVID-19 cases have often used whole blood samples from patients at various timepoints following hospitalization and do not control for neutrophil proportions, numbers, or the presence of contaminating cells such as monocytes, which can express typical neutrophil-associated genes while also being more transcriptionally active. Thus, the differential gene expression of neutrophils associated with severe disease may be confounded by technical issues or reflect different disease stages in such studies. From our study, it is clear neutrophil gene expression of primary granule proteins can vary between genetic backgrounds and this heterogeneity can contribute heavily to pulmonary pathology during a respiratory coronavirus infection.

A previous study using a chimeric virus model showed that mortality in MHV-infected A/J mice was not strongly associated with viral load but instead with inflammation (7). Neutrophils in A/J mice may be more prone to NET release or cause more collateral damage with degranulation due to their higher primary granule protein reserves compared to their B6 counterparts, leading to lung injury and beginning an inflammatory cascade that fails to target the viral pathogen. Indeed, despite high viral loads in the A/J mice, the canonical antiviral cytokines IFNß and IFNγ were significantly increased compared to their B6 counterparts only at day 7 PI. IL-1α levels, most commonly associated with epithelial cell or macrophage necrosis in the lungs, were elevated at least by day 3 and through day 7 of infection in A/J lungs indicating high necrotic damage prior to and during the development of an antiviral response (48). Interestingly, IL-1ß was only elevated in A/J lungs at day 3 and not day 7, coinciding with the neutrophil influx. Neutrophils have been shown to be critical for pro-IL-1ß cleavage after its release by alveolar macrophages in an influenza model, and can additionally release mature IL-1ß during bacterial pneumonia resulting in increased neutrophilia and innate inflammation (49, 50). This inflammatory response could damage barrier cells, promoting viral dissemination. Additionally, IL-1 driven signaling may antagonize or delay type I IFN responses; delayed type I IFN responses can promote viral replication and induce hyperinflammation in COVID-19 (51, 52).

The role of neutrophils in mediating host damage and pathogen clearance in viral respiratory infections is still poorly understood. It has become clear that these cells exhibit a vast functional heterogeneity with functions ranging from immunoregulation to wound repair, as opposed to their classical characterization as simple antimicrobial phagocytes with an extensive capacity for collateral damage through their granule armaments (53). In support of the complexity of neutrophil subtypes, we have recently reported on transcriptomic-based neutrophil heterogeneity within human subjects with and without SARS-CoV-2 infection. The neutrophil heterogeneity between mouse strains as demonstrated here could be used to further elucidate their roles in different viral infections. In other viral respiratory infection models, an early neutrophil influx has been found to be required to limit disease severity. For example, in a model of human metapneumovirus infection in BALB/c mice, 1A8 antibody-mediated neutrophil depletion pre-infection exacerbated lung damage and increased morbidity (54). In B6 mice, neutrophil depletion has been observed to have negative effects on lung damage and survival in influenza infection models. Pre-infection neutrophil depletion in a A/HKx31;H3N2 influenza infection model leads to increased lung damage and complete mortality with a normally mild infection dose, while depletion quickens mortality with a lethal dose of highly virulent A/PR/8/34;H1N1 (PR8) virus (55, 56). The PR8 infection model in B6 mice is associated with a highly neutrophilic lung leukocyte response compared to the HKx31 infection model, and interestingly an incomplete depletion of neutrophils with a lethal PR8 dose resulted in significantly improved survival without affecting viral loads (57). These results suggest that different subsets of these cells may play important regulatory roles beyond killing pathogens, and an appropriate balance of functional subtypes of neutrophils is required to limit immunopathogenicity. The A/J mouse strain exhibits a marked susceptibility to a mouse-adapted A/HK/1/68;H3N2 influenza strain as well as extreme susceptibility to PR8 flu virus compared to B6 mice (58–60). As in the A/J MHV-1 model, the A/J H3N2 model is marked by increased inflammatory cytokine production and neutrophilic lung infiltration compared to B6 mice, and the neutrophil influx may similarly drive lung damage seen in flu-infected A/J mice (25, 59). Neutrophil depletions have not been attempted in an A/J influenza model but may help further elucidate how an early neutrophilic lung leukocyte influx affects the severity of a respiratory viral infection. Due to additional differences in granule composition observed here, however, the effects of complete or partial neutrophil depletion in the A/J mouse may vary from those observed in B6 infection models.

This model demonstrates that neutrophils have strain-dependent effects on lung pathology during a murine model of respiratory coronaviral infection. In summary, our data suggests that there are inborn differences in neutrophil function and these cells have heterogenous roles in infection that vary between mouse strains. Whereas neutrophils in A/J mice contributed heavily to observed pulmonary pathology, the depletion of these cells from B6 mice had minimal effect on lung injury during MHV-1 infection. These results reinforce the fact that neutrophils are a phenotypically heterogenous cell type and may similarly vary between human individuals as they vary between strains of mice. Further studies are necessary to understand the contribution of these phenotypic differences to disease pathogenesis and the poorly understood mechanisms that regulate their differentiation.

## Data availability statement

The original contributions presented in the study are included in the article/**Supplementary Material**. Further inquiries can be directed to the corresponding author.

## Ethics statement

The animal study was reviewed and approved by Veterans Affairs Institutional Animal Care and Use Committee.

## Author contributions

HG and MW conducted the research and wrote the manuscript. KC and DG assisted in the research and edited the manuscript. JD supervised the work and edited the manuscript. All authors contributed to the article and approved the submitted version.
