# Supplementary figures and images for: Neutrophils drive pulmonary vascular leakage in MHV-1 infection of susceptible A/J mice
Source: Front Immunol. 2023 Jan 6;13:1089064. doi: 10.3389/fimmu.2022.1089064 (PMC9853883; doi:10.3389/fimmu.2022.1089064)

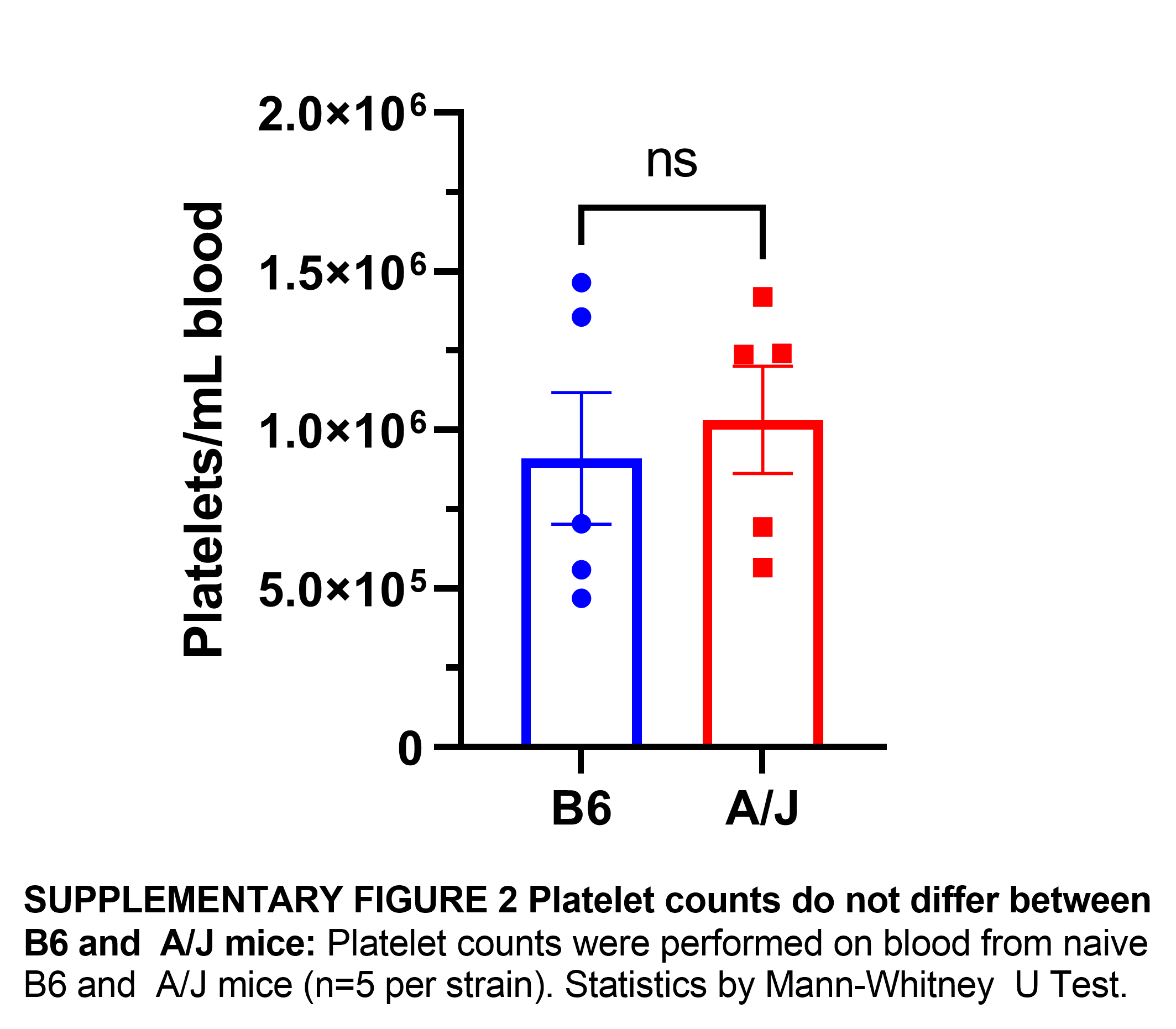

Supplement: Supplementary file 2 [file Image_2.tif]

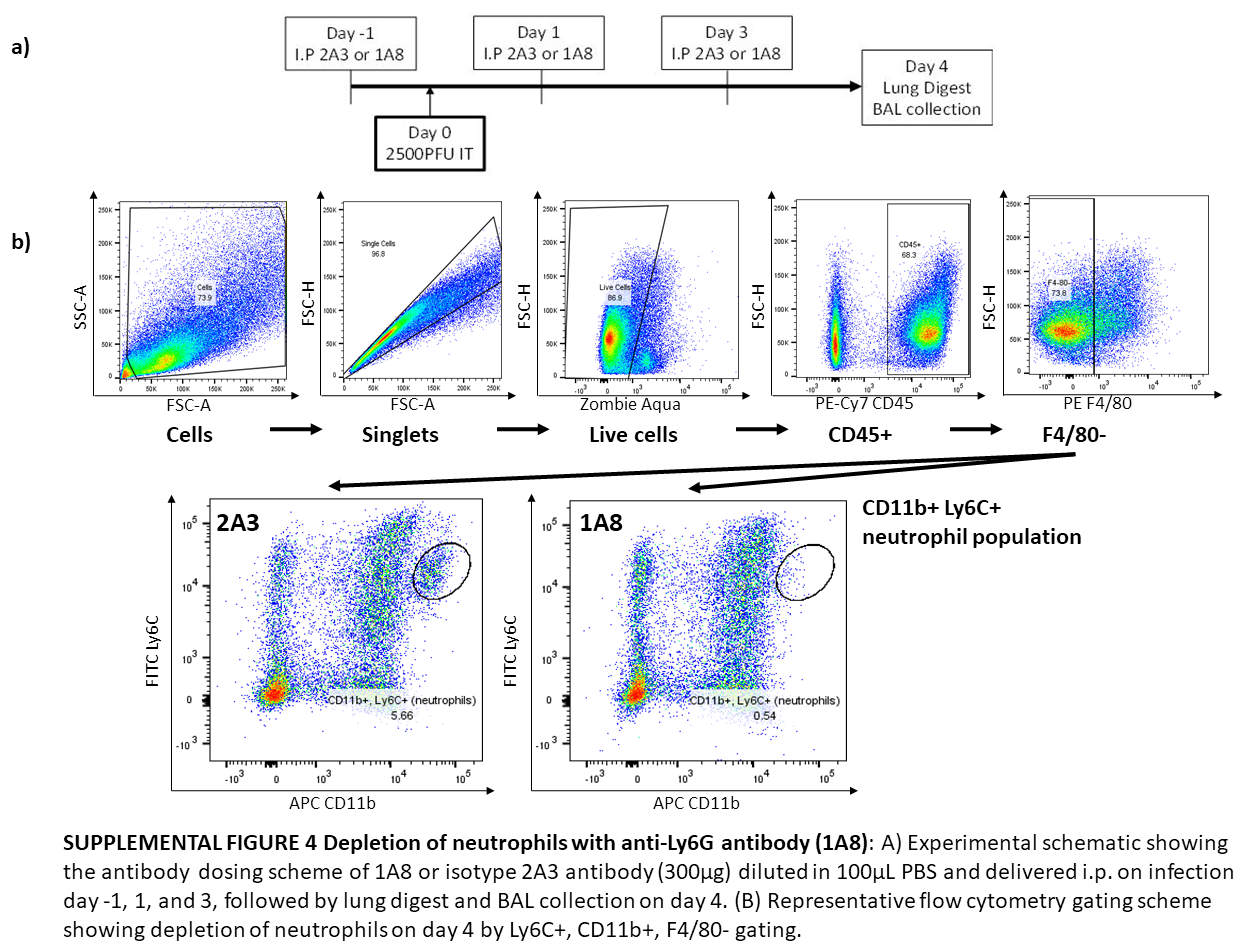

Supplement: Supplementary file 4 [file Image_4.png]
